# High-Dose Methotrexate in Children and Young Adults With ALL and Lymphoblastic Lymphoma: Results of the Randomized Phase III Study UKALL 2011

**DOI:** 10.1200/JCO-24-01851

**Published:** 2025-04-07

**Authors:** Amy A. Kirkwood, Nicholas Goulden, John Moppett, Sujith Samarasinghe, Rachael Hough, Clare Rowntree, Sarah Lawson, Pam Kearns, Anna Lawson, Ajay Vora

**Affiliations:** ^1^ CR UK & UCL Cancer Trials Centre, UCL Cancer Institute, UCL, London; ^2^ Great Ormond Street Hospital, London; ^3^ Bristol Children's Hospital, Bristol; ^4^ University College Hospital, London; ^5^ University Hospital of Wales, Cardiff; ^6^ Birmingham Children's Hospital, Birmingham; ^7^ Cancer Research Cancer Trials Unit, University of Birmingham, Birmingham, United Kingdom

## Abstract

**PURPOSE:**

UKALL 2011 randomly assigned children and young adults (younger than 25 years) with ALL or lymphoblastic lymphoma. The aims were to reduce induction toxicity (randomization 1 [R1]), CNS relapse risk (randomization 2 [R2]–interim maintenance [R2IM]), and maintenance morbidity (R2pulses).

**METHODS:**

R1 compared induction dexamethasone (dex) for 28 days (6 mg/m^2^; standard) with 14 days (10 mg/m^2^; short). R2 was a factorial randomization resulting in four arms: high-dose methotrexate (HDM) with pulses, HDM without pulses, standard interim maintenance (SIM) with pulses (standard of care), and SIM without pulses. The primary end points were reduction in steroid-related toxicity (R1), CNS relapse rate (CNSR, R2IM), and bone marrow relapse rate (BMR, R2pulses; ALL only, noninferiority margin 5%). Event-free survival (EFS) was an additional primary end point for both randomizations.

**RESULTS:**

Of 2,750 eligible patients registered between April 2012 and December 2018, 1,902 were randomly assigned to R1 and 1,570 to R2. Median follow-up is 99 (R1) and 87 months (R2). There were no differences in steroid-related toxicity between short and standard dex (23.8% *v* 25.5%; *P* = .41) and CNSR between SIM and HDM (0.98 [95% CI, 0.65 to 1.49]; *P* = .94; 5-year rates: SIM 5.3% and HDM 5.5%). EFS was no different between R1 and R2IM arms. BMR in the no pulses arm was noninferior (+1.7% increase at 5 years [95% CI, –1.5 to 4.1]; hazard ratio [HR], 1.19 [95% CI, 0.87 to 1.62]; *P* = .27). Although the EFS in the no pulses arm was inferior (1.34 [95% CI, 1.05 to 1.73]; *P* = .021), this was not significant for relapse (HR, 1.24 [95% CI, 0.96 to 1.62]; *P* = .10).

**CONCLUSION:**

Shorter duration of induction dex does not reduce steroid-related toxicity and HDM does not improve CNSR within a UKALL treatment backbone. Omission of pulses is noninferior for BMR.

## INTRODUCTION

With long-term relapse-free survival of over 85%,^1^ the challenge for contemporary clinical trials is to sustain that rate of improvement and to address the problem of immediate and long-term toxicity of treatment.^2^ In a previous UK trial, UKALL 2003,^3^ the risk of relapse at 5 years had nearly halved to 10% compared with its predecessor,^4^ but the risk of death due to treatment complications remained unchanged at around 5%. Another 10% of patients experienced serious nonfatal treatment-related side effects, which had a considerable impact on quality of life (QoL),^5^ burden of care, and longer-term health outcomes.^6^ In the successor randomized clinical trial, UKALL 2011, we tested two interventions designed primarily to reduce toxicity while maintaining efficacy and one to reduce the risk CNS relapse. We also adopted a nonrandomized reduction in the intensity of the treatment backbone compared with our previous trial and refined the risk stratification model to identify a group at very high risk of relapse. Here, we report the outcome for patients in the trial overall and the results of the randomized interventions.

**CONTEXT**

**Key Objective**
The primary aim of this randomized trial was to reduce toxicity and improve CNS outcomes.
**Knowledge Generated**
The results were confounded by an interaction between the randomizations, but we found that dexamethasone given for 14 days is no less toxic than that given throughout induction, high-dose methotrexate is no better than intensive intrathecal therapy in preventing CNS relapses, and omission of maintenance pulses is noninferior for bone marrow relapse.
**Relevance *(S. Lentzsch)***
These findings suggest that current treatment protocols for ALL should remain unchanged, while reductions in treatment-related mortality underscore the benefits of optimized supportive care.*
*Relevance section written by *JCO* Associate Editor Suzanne Lentzsch, MD, PhD.

## METHODS

### Study Design and Participants

UKALL 2011 was a multicenter investigator-initiated umbrella protocol comprising three randomized open-label phase III questions. Randomization 1 (R1) aimed to reduce toxicity by replacing standard dose dexamethasone (dex; standard) with a higher dose given over a shorter duration (short). Randomization 2 (R2) was a factorial randomization aimed to reduce the risk of CNS relapse (R2-interim maintenance [R2IM]) by replacing the standard interim maintenance (SIM) block of therapy (SIM) with a high-dose methotrexate (HDM) containing block and to improve QoL in the maintenance phase by the removal of dex and vincristine (VCR) pulses (R2pulses). This latter question was a noninferiority design that aimed to preserve a similar rate of bone marrow (BM) relapse.

The trial recruited children and young adults with Philadelphia-negative ALL and lymphoblastic lymphoma (LBL) diagnosed in the United Kingdom and Ireland except those younger than 1 year or with mature B-ALL. The upper age limit of entry was to the day before the 25th birthday. Patients with Down syndrome were eligible for R1 but not R2 as they are known to have an increased risk of HDM toxicity.

### Procedures

#### Risk Stratification and Treatment

Initial risk allocation was based on presenting characteristics, leukemia immunophenotype, and cytogenetics. Subsequently, patients were stratified by BM minimal residual disease (MRD) at end of induction and consolidation to MRD low-risk, intermediate-risk, and high-risk groups. See the Data Supplement (Fig S1 and Methods S1, online only) for details of diagnosis, MRD measurement, stratification, and treatment backbone including CNS-directed therapy.

#### Randomized Interventions

R1 randomly assigned patients at the start of induction to dex 5 mg/m^2^ twice daily for 14 days (continuous in age <10 years and discontinuous week on/week off in age ≥10 years; short) or 3 mg/m^2^ twice daily for 29 days followed by a 7-day wean (standard).

SIM was for 2 months with oral mercaptopurine (MP) 75 mg/m^2^ once daily and oral methotrexate (MTX) 20 mg/m^2^ once weekly, once monthly pulses and single intrathecal MTX in regimens A and B, and five doses of escalating intravenous MTX (Capizzi) + VCR 1.5 mg/m^2^ + two doses of pegylated asparaginase in regimen C. HDM was given at a dose of 5 g/m^2^ × four doses 2 weeks apart with folinic acid rescue, low-dose oral MP (25 mg/m^2^), and two doses of pegylated asparaginase (regimen C only). Pulses were a single dose of VCR and 5 days of dex (3 mg/m^2^ twice a day) given once monthly during maintenance cycles.

### Statistical Analysis

The primary end point for R1 was a composite end point of serious steroid-related adverse event (AE)/serious adverse events (Common Terminology Criteria for Adverse Events V4.0 grade ≥3 or above) or death during induction not primarily related to ALL/LBL; we aimed to reduce this from 8% to 4.8% (risk ratio 0.6); 2,376 patients would give 89% power to detect this (two-sided 5% α). For R2IM, the primary end point was the rate of CNS relapse believed to be 4% at 5 years, which we aimed to reduce to 1.5% (hazard ratio [HR], 0.37). One thousand eight hundred sixteen randomly assigned patients would give 90% power to detect this (two-sided 5% α). R2pulses aimed to show that removing pulses would be noninferior in terms of BM relapse (thought to be 10%-15% at 5 years) for patients with ALL and would improve QoL at 18 months (to be presented in a separate publication). One thousand seven hundred fifty-seven patients with ALL (available from 1,816 R2 randomly assigned patients) would give over 85% power to exclude a difference of 5% or more with a one-sided 2.5% α. Event-free survival (EFS; events: relapse, death or second malignancy) was considered a primary end point for all randomizations and for the overall trial cohort but was not powered to detect any prespecified differences. The primary efficacy analyses are based on the modified intention-to-treat (ITT) population (excluding those found to be ineligible after R1/registration) except for the noninferiority analysis of R2pulses and toxicity, which are based on patients who started their randomized allocation (per-protocol).

For full details on secondary end points and methods of analysis, see the Data Supplement (Methods S2).

### Ethics Statement

The protocol was approved by the Central London Research Ethics Committee. Written informed consent was obtained from carers and patients as appropriate. The trial was monitored by an independent data monitoring committee that reviewed safety and efficacy data at least annually.

## RESULTS

**FIG 1.**
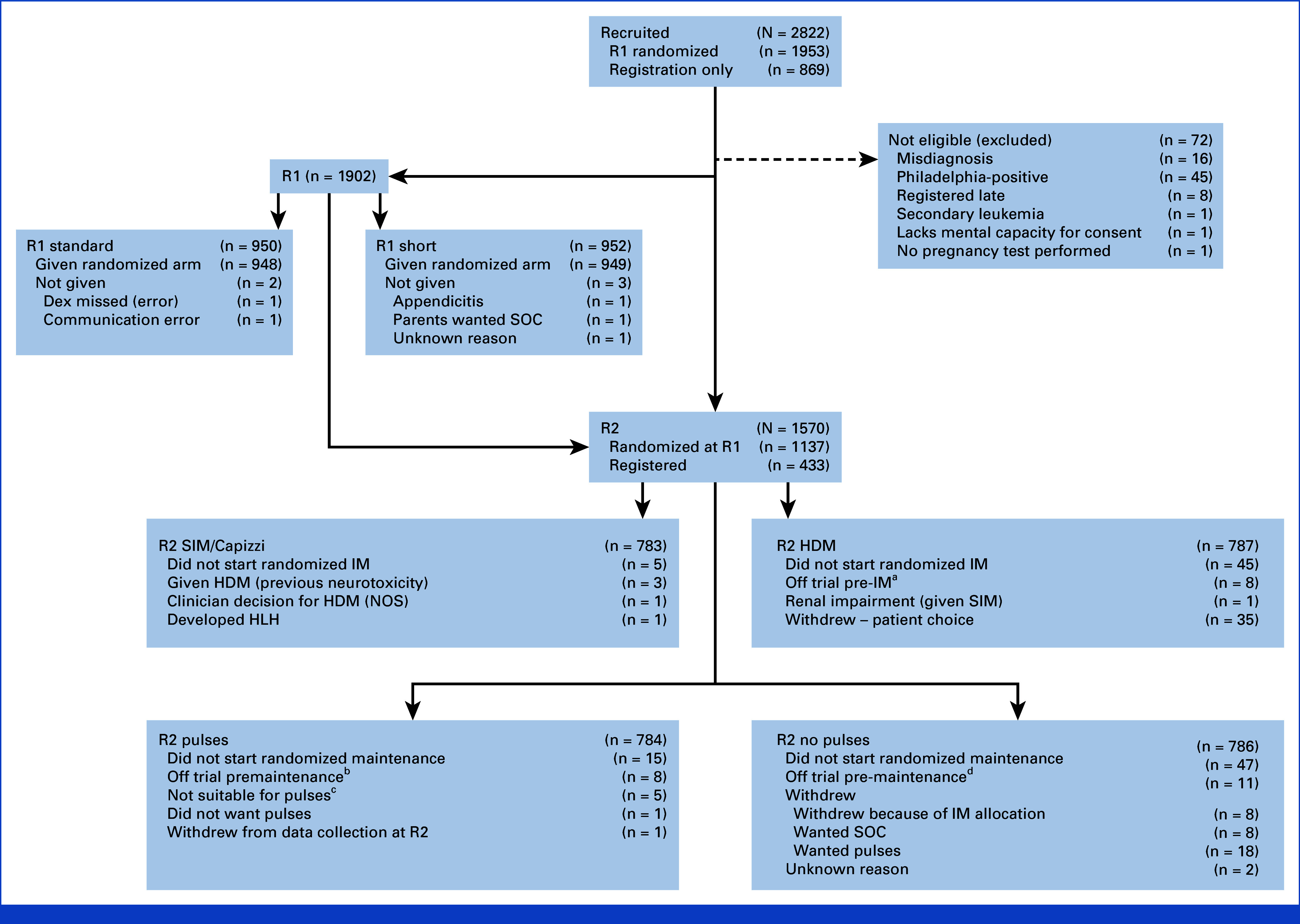
CONSORT diagram. ^a^Died (N = 1), relapsed (N = 3), and AEs (N = 4). ^b^Died (N = 1), relapsed (N = 4), and treatment change because of higher-risk disease (N = 3). ^c^Osteonecrosis (N = 1), vincristine neurotoxicity (N = 2), diabetes (N = 1), and neuropathy/cardiac failure and foot ulcer (N = 1).^d^Relapsed (N = 8), developed HLH (N = 1), off trial because of AEs (N = 1), and unknown reason (N = 1). AE, adverse event; Dex, dexamethasone; HDM, high-dose methotrexate; HLH, hemophagocytic lymphohistiocytosis; IM, interim maintenance; NOS, not otherwise specified; R1, randomization 1; R2, randomization 2; SIM, standard interim maintenance; SOC, standard of care.

Two thousand eight hundred twenty-two patients were registered on to the trial from April 26, 2012, to December 31, 2018; 72 were ineligible, leaving 2,750 included in these results (Fig 1). On the recommendation of the International Data Monitoring Committee, R1 closed in April 2017 after recruiting 1,902 patients because of concern about excess treatment-related deaths in the short dex arm and futility of the primary end point. Subsequent patients were registered at diagnosis and offered the second randomizations (R2), which recruited 1,570 patients, of whom 433 were randomly assigned after closure of R1. Characteristics of patients in the trial overall and the randomizations are shown in Table 1 (R1) and Table 2 (R2) and were well balanced between the arms. Follow-up is to December 2, 2024, with median follow-up of 99.1 months for R1 (IQR, 86.6-112.7) and 87.0 months (IQR, 70.8-106.3) for R2.

**TABLE 1.** Characteristics of Patients in Trial Overall and R1

Characteristic	Standard Dex (n = 950)	Short Dex (n = 952)	All (N = 2,750)
Age,^a^ years, median (IQR)	5.0 (3.0-11.0)	5.0 (3.0-11.0)	5 (3.0-11.0)
Range	1-25	1-24	1-25
Age (group), No. (%)			
Under 10	678 (71.4)	673 (70.7)	1,964 (71.4)
10-15	158 (16.6)	164 (17.2)	471 (17.1)
16 and over	114 (12.0)	115 (12.1)	315 (11.5)
Ethnicity, No. (%)			
Asian—Bangladeshi	6 (0.7)	2 (0.2)	13 (0.5)
Asian—Pakistani	25 (3.1)	22 (2.7)	61 (2.6)
Asian—Indian	10 (1.2)	21 (2.5)	57 (2.4)
Black—Caribbean	1 (0.1)	2 (0.2)	4 (0.2)
Black—African	10 (1.2)	6 (0.7)	19 (0.8)
Chinese	3 (0.4)	3 (0.4)	7 (0.3)
Mixed—White and Black African	20 (2.4)	8 (1.0)	42 (1.8)
Mixed—White and Black Caribbean	5 (0.6)	4 (0.5)	12 (0.5)
Other Asian background	21 (2.6)	15 (1.8)	51 (2.1)
Other Black background	3 (0.4)	2 (0.2)	8 (0.3)
Other ethnic	15 (1.8)	14 (1.7)	37 (1.6)
Other mixed background	16 (2.0)	16 (1.9)	52 (2.2)
White	684 (83.5)	713 (86.1)	2,017 (84.7)
Missing	131	124	370
Sex, No. (%)			
Male	537 (56.5)	543 (57.0)	1,579 (57.4)
Female	413 (43.5)	409 (43.0)	1,171 (42.6)
Down's syndrome, No. (%)			
No	926 (97.5)	933 (98.0)	2,691 (97.9)
Yes	24 (2.5)	19 (2.0)	59 (2.1)
CNS status, No. (%)			
1	838 (93.4)	834 (93.2)	2,421 (92.9)
2	38 (4.2)	32 (3.6)	115 (4.4)
3	21 (2.3)	29 (3.2)	69 (2.6)
Missing	53	57	145
Did patient have a traumatic lumbar puncture? No. (%)			
No	780 (85.1)	701 (87.4)	1,581 (86.2)
Yes	137 (14.9)	116 (12.6)	253 (13.8)
Traumatic (without blasts)	105 (77.2)	93 (83.0)	198 (79.8)
Traumatic (with blasts)	31 (22.8)	19 (17.0)	50 (20.2)
Missing	1	4	5
Missing	33	35	916
High-risk cytogenetics, No. (%)			
No	889 (95.9)	879 (95.0)	2,529 (95.1)
Yes	38 (4.1)	46 (5.0)	131 (4.9)
iAMP21	8 (23.5)	16 (37.2)	38 (33.0)
t(17;19)	4 (11.8)	1 (2.3)	6 (5.2)
*KMT2A* rearrangement	19 (55.9)	18 (41.9)	53 (46.1)
Near haploidy	1 (2.9)	4 (9.3)	9 (7.8)
Low hypodiploidy	2 (5.9)	4 (9.3)	9 (7.8)
Missing	4	3	16
Missing/patient does not have an induction form	23	27	90
Disease/immunophenotype, No. (%)			
B-ALL	793 (83.5)	798 (83.8)	2,275 (82.7)
T-ALL	108 (11.4)	106 (11.1)	326 (11.9)
B-LBL	17 (1.8)	9 (0.9)	37 (1.3)
T-LBL	32 (3.4)	39 (4.1)	112 (4.1)

ALL	Standard Dex (n = 901)	Short Dex (n = 904)	All (N = 2,601)
WCC, median (IQR)	12.2 (4.7-42.0)	11.0 (4.6-42.6)	12.15 (4.7-43.9)
Range	0.1-817	0.4-1,000	0.1-1,800
WCC group, No. (%)			
<50	707 (78.5)	705 (78.2)	2,019 (77.7)
≥50	194 (21.5)	197 (21.8)	579 (22.3)
Missing	0	2	3
NCI risk group, No. (%)			
Standard	516 (57.3)	514 (57.0)	1,496 (57.6)
High	385 (42.7)	388 (43.0)	1,103 (42.4)
Missing	0	2	2

NOTE. R1 (short *v* standard dex) was stratified by disease type (ALL/LBL), sex, age, and WCC.

Abbreviations: dex, dexamethasone; LBL, lymphoblastic lymphoma; NCI, National Cancer Institute; R1, randomization 1; R2, randomization 2; WCC, white cell count.

^a^ Includes two patients who were age 25 years at random assignment, but age 24 years at diagnosis as per protocol inclusion criteria.

**TABLE 2.** Characteristics of Patients in R2 Arms

Characteristic	SIM + Pulses (n = 391)	SIM, No Pulses (n = 392)	HDM + Pulses (n = 393)	MTX, No Pulses (n = 394)	All (N = 1,570)
Age,^a^ years, median (IQR)	5.0 (3.0-11.0)	5.0 (3.0-11.0)	5.0 (3.0-11.0)	5.0 (3.0-11.0)	5 (3.0-11.0)
Range	1-25	1-25	1-24	1-24	1-25
Age (group), No. (%)					
Under 10	280 (71.6)	281 (71.7)	268 (68.2)	274 (69.5)	1,103 (70.3)
10-15	65 (16.6)	59 (15.1)	75 (19.1)	73 (18.5)	272 (17.3)
16 and over	46 (11.8)	52 (13.3)	50 (12.7)	47 (11.9)	195 (12.4)
Ethnicity, No. (%)					
Asian—Bangladeshi	2 (0.6)	2 (0.6)	1 (0.3)	2 (0.6)	7 (0.5)
Asian—Pakistani	10 (2.9)	7 (2.1)	7 (2.0)	9 (2.6)	33 (2.4)
Asian—Indian	8 (2.4)	5 (1.5)	11 (3.2)	6 (1.7)	30 (2.2)
Black—African	1 (0.3)	1 (0.3)	3 (0.9)	3 (0.9)	8 (0.6)
Chinese	0	1 (0.3)	2 (0.6)	2 (0.6)	5 (0.4)
Mixed—White and Black African	8 (2.4)	5 (1.5)	5 (1.4)	3 (0.9)	21 (1.5)
Mixed—White and Black Caribbean	2 (0.6)	2 (0.6)	1 (0.3)	0	5 (0.4)
Other Asian background	9 (2.6)	9 (2.6)	10 (2.9)	4 (1.2)	32 (2.3)
Other Black background	1 (0.3)	1 (0.3)	1 (0.3)	1 (0.3)	4 (0.3)
Other ethnic	8 (2.4)	6 (1.8)	3 (0.9)	6 (1.7)	23 (1.7)
Other mixed background	5 (1.5)	9 (2.6)	5 (1.4)	13 (3.8)	32 (2.3)
White	286 (84.1)	292 (85.9)	296 (85.8)	296 (85.8)	1,170 (85.4)
Missing	51	52	48	49	200
Sex, No. (%)					
Male	226 (57.8)	230 (58.7)	231 (58.8)	231 (58.6)	918 (58.5)
Female	165 (42.2)	162 (41.3)	162 (41.2)	163 (41.4)	652 (41.5)
Down syndrome, No. (%)					
No	391 (100.0)	392 (100.0)	393 (100.0)	394 (100.0)	1,570 (100.0)
CNS status, No. (%)					
1	342 (93.4)	353 (93.9)	348 (91.8)	340 (92.4)	1,383 (92.9)
2	13 (3.6)	14 (3.7)	17 (4.5)	18 (4.9)	62 (4.2)
3	11 (3.0)	9 (2.4)	14 (3.7)	10 (2.7)	44 (3.0)
Missing	25	16	14	26	81
Did patient have a traumatic lumbar puncture? No. (%)					
No	239 (87.5)	241 (87.6)	233 (85.0)	240 (87.6)	953 (87.0)
Yes	34 (12.5)	34 (12.4)	41 (15.0)	34 (12.5)	143 (13.0)
Traumatic (without blasts)	29 (85.3)	26 (81.2)	34 (82.9)	29 (85.3)	118 (83.7)
Traumatic (with blasts)	5 (14.7)	6 (18.8)	7 (17.1)	5 (14.7)	23 (16.3)
Missing	0	2	0	0	2
Missing	118	117	119	120	474
High-risk cytogenetics,^b^ No. (%)					
No	362 (96.3)	360 (94.7)	361 (93.5)	371 (96.4)	1,454 (95.2)
Yes	14 (3.7)	20 (5.3)	25 (6.5)	14 (3.6)	73 (4.8)
iAMP21	5 (41.7)	7 (35.0)	6 (27.3)	4 (40.0)	22 (34.4)
t(17;19)	0	0	2 (9.1)	0	2 (3.1)
*KMT2A* rearrangement	7 (58.3)	8 (40.0)	10 (45.5)	5 (50.0)	30 (46.9)
Near haploidy	0	2 (10.0)	2 (9.1)	0	4 (6.2)
Low hypodiploidy	0	3 (15.0)	2 (9.1)	1 (10.0)	6 (9.4)
Missing	2	0	3	4	9
Missing	15	12	7	9	43
Disease/immunophenotype, No. (%)					
B-ALL	329 (84.1)	327 (83.4)	326 (83.0)	331 (84.0)	1,313 (83.6)
T-ALL	41 (10.5)	44 (11.2)	45 (11.5)	41 (10.4)	171 (10.9)
B-LBL	3 (0.8)	7 (1.8)	7 (1.8)	3 (0.8)	20 (1.3)
T-LBL	18 (4.6)	14 (3.6)	15 (3.8)	19 (4.8)	66 (4.2)
R1, No. (%)					
Standard dex	140 (35.8)	141 (36.0)	142 (36.1)	139 (35.3)	562 (35.8)
Short dex	143 (36.6)	143 (36.5)	144 (36.6)	145 (36.8)	575 (36.6)
No R1	108 (27.6)	108 (27.6)	107 (27.2)	110 (27.9)	433 (27.6)
Regimen after induction, No. (%)					
A	131 (33.5)	124 (31.6)	116 (29.5)	126 (32.0)	497 (31.7)
B	87 (22.3)	84 (21.4)	95 (24.2)	93 (23.6)	359 (22.9)
C	173 (44.2)	184 (46.9)	182 (46.3)	175 (44.4)	714 (45.5)

ALL	SIM + Pulses (n = 370)	SIM, No Pulses (n = 371)	HDM + Pulses (n = 371)	MTX, No Pulses (n = 372)	All (N = 1,484)
WCC, median (IQR)	11.4 (4.5-41.0)	11.2 (4.3-34.0)	12.2 (4.4-40.2)	12.0 (4.8-44.4)	11.7 (4.5-41.0)
Range	0.6-440	0.1-817	0.5-756.9	0.3-578.4	0.1-817
WCC group, No. (%)					
<50	290 (78.4)	293 (79.2)	299 (80.6)	288 (77.4)	1,170 (78.9)
≥50	80 (21.6)	77 (20.8)	72 (19.4)	84 (22.6)	313 (21.1)
Missing	0	1	0	0	1
NCI risk group, No. (%)					
Standard	217 (58.6)	211 (57.0)	210 (56.6)	205 (55.1)	843 (56.8)
High	153 (41.4)	159 (43.0)	161 (43.4)	167 (44.9)	640 (43.2)
Missing	0	1	0	0	1
MRD (R2), No. (%)					
MRD low risk	202 (54.6)	188 (50.7)	186 (50.1)	199 (53.5)	775 (52.2)
MRD intermediate risk	148 (40.0)	163 (43.9)	162 (43.7)	151 (40.6)	624 (42.0)
MRD no result	20 (5.4)	20 (5.4)	23 (6.2)	22 (5.9)	85 (5.7)

NOTE. R2 was a factorial randomisation to HDM or SIM and to pulses or no pulses in maintenance. This second randomization was stratified as per R1 (disease type [ALL/LBL], sex, age, and WCC) with two additional factors: MRD (low or intermediate risk) and R1 steroid (standard or short).

Abbreviations: dex, dexamethasone; HDM, high-dose methotrexate; LBL, lymphoblastic lymphoma; MRD, minimal residual disease; MTX, methotrexate; NCI, National Cancer Institute; R1, randomization 1; R2, randomization 2; SIM, standard interim maintenance; WCC, white cell count.

^a^ Includes two patients who were age 25 years at random assignment, but age 24 years at diagnosis, as per protocol inclusion criteria.

### Overall Outcomes

**FIG 2.**
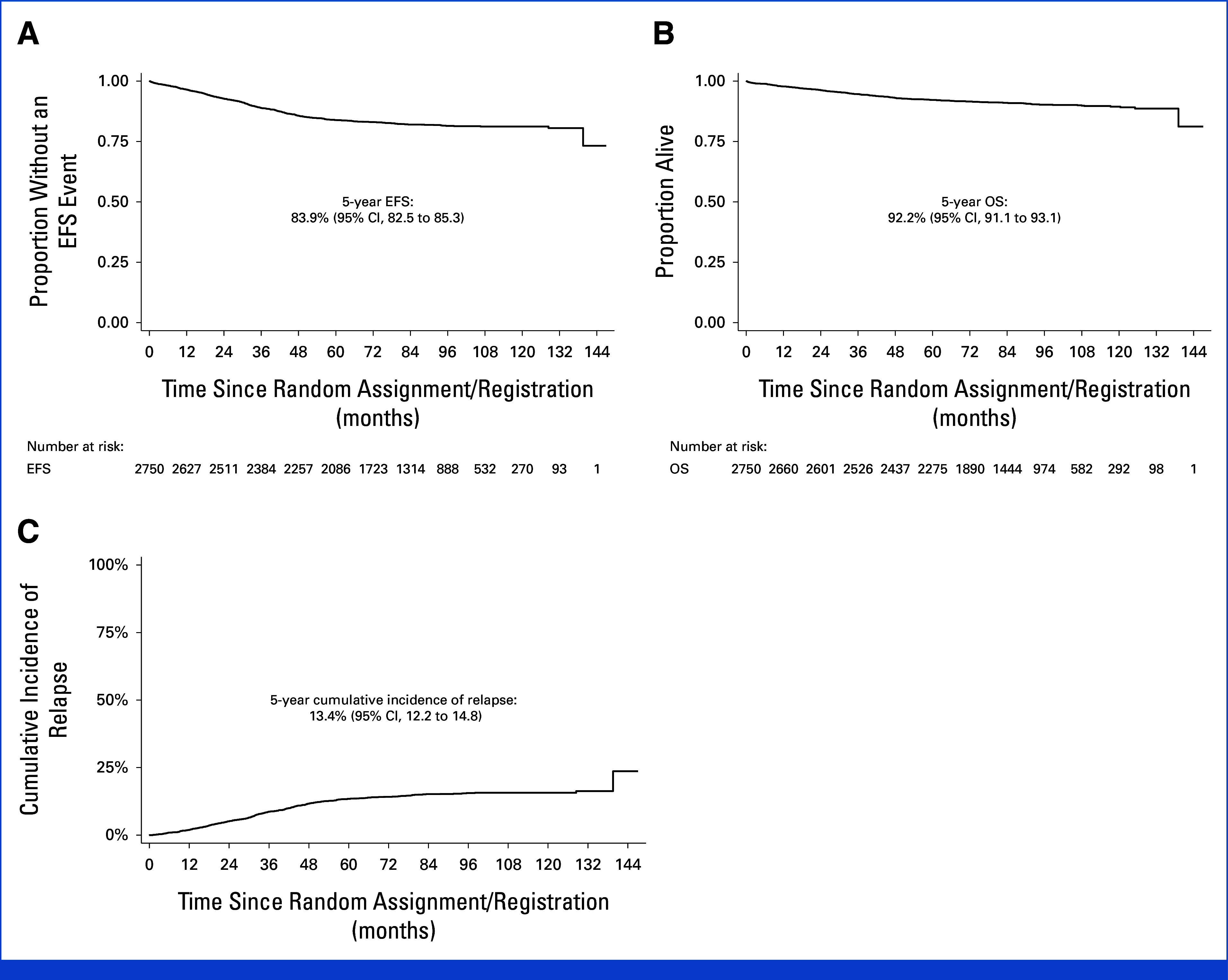
(A) EFS, (B) OS, and (C) relapse risk in trial overall. EFS, event-free survival; OS, overall survival.

In the trial overall, there have been 481 EFS events and 251 deaths giving a 5-year EFS of 83.9% (95% CI, 82.5 to 85.3) and OS of 92.2% (95% CI, 91.1 to 93.1; Fig 2). The 5-year cumulative relapse risk was 13.4% (95% CI, 12.2 to 14.8). The risk of BM relapse (ALL only) was 10.0% (95% CI, 8.9 to 11.2), any CNS relapse 5.1% (95% CI, 4.3 to 6.0), and isolated CNS relapse 3.2% (95% CI, 2.6 to 4.0; Table 3). Seventy patients (2.5% of trial population) received an SCT in first remission for primary refractory disease, 19 relapsed, and 16 died without relapse reported; the 5-year EFS from SCT was 46.3% (95% CI, 33.8 to 57.9).

**TABLE 3.** Event Rates and Randomized Comparisons by Arm and in the Trial Overall

Event	Standard	Short	SIM	HDM	Pulses	No Pulses	All Patients
Induction failure,^a^ No. (%)	34 (3.6)	42 (4.4)	4 (0.5)	10 (1.3)	6 (0.8)	8 (1.0)	114 (4.2)
	*P* = .41						
Induction TRM, No. (%)	6 (0.6)	10 (1.1)	—	—	—	—	17 (0.6)
	*P* = .45						
High-risk MRD at week 14 (TP2), No. (%)	5 (0.5)	8 (0.8)	—	—	—	—	15 (0.6)
	*P* = .58						
EFS							
Events, No./n	165/950	196/952	137/783	133/787	117/784	153/786	481/2,750
5-year EFS rate, % (range)	83.9 (81.4-86.1)	81.7 (78.1-84.0)	84.0 (81.2-86.4)	84.8 (82.1-87.2)	86.7 (84.1-88.9)	82.1 (79.2-84.7)	83.9 (82.5-85.3)
HR (95% CI)	1.21 (0.98 to 1.49) *P* = .069		0.97 (0.77 to 1.24) *P* = .83		1.34 (1.06 to 1.71) *P* = .016		—
OS							
Deaths, No./n	100/950	103/952	66/783	59/787	52/784	73/786	251/2,750
5-year OS rate, % (range)	91.5 (89.5-93.1)	90.8 (88.7-92.5)	93.3 (91.3-94.9)	93.4 (91.4-94.9)	94.7 (92.9-96.1)	91.9 (89.8-93.7)	92.2 (91.1-93.1)
HR (95% CI)	1.04 (0.79 to 1.37) *P* = .78		0.89 (0.63 to 1.27) *P* = .53		1.42 (1.00 to 2.03) *P* = .050		—
Relapse							
Relapse, No./n	129/950	169/952	125/783	122/787	110/784	137/786	403/2,750
5-year cumulative incidence, % (range)	12.6 (10.6-14.9)	15.7 (13.5-18.2)	14.5 (12.2-17.2)	14.3 (12.0-16.9)	12.7 (10.5-15.2)	16.1 (13.7-18.9)	13.4 (12.2-14.8)
HR (95% CI)	1.34 (1.07 to 1.69) *P* = .012		0.98 (0.77 to 1.26) *P* = .89		1.27 (0.99 to 1.63) *P* = .062		—
BM relapse (ALL only)^b^							
BM relapse, No./n	97/901	121/904	92/783	83/787	76/729	86/701	291/2,601
5-year cumulative incidence, % (range)	9.8 (8.0-11.9)	11.4 (9.5-13.7)	11.2 (9.1-13.8)	9.6 (7.7-12.0)	9.0 (7.1-11.4)	11.1 (9.0-13.7)	10.0 (8.9-11.2)
HR (95% CI)	1.26 (0.96 to 1.65) *P* = .089		0.89 (0.66 to 1.19) *P* = .42		1.19 (0.87 to 1.62) *P* = .27		—
5-year difference					+1.7% (–1.5% to 4.1%)		
CNS relapse							
CNS relapse, No./n	44/950	65/952	46/783	45/787	39/784	52/786	148/2,750
5-year cumulative incidence, % (range)	4.4 (3.3-5.9)	6.3 (4.9-8.1)	5.3 (3.9-7.2)	5.5 (4.1-7.4)	4.7 (3.4-6.4)	6.2 (4.7-8.1)	5.1 (4.3-6.0)
HR (95% CI)	1.50 (1.02 to 2.19) *P* = .038		0.98 (0.65 to 1.49) *P* = .94		1.35 (0.89 to 2.04) *P* = .16		—
Isolated CNS relapse							
Isolated CNS relapse, No./n	22/950	41/952	26/783	32/787	23/784	35/786	90/2,750
5-year cumulative incidence, % (range)	2.3 (1.5-3.4)	4.2 (3.1-5.6)	3.1 (2.1-4.6)	4.1 (2.9-5.8)	3.0 (2.0-4.4)	4.2 (3.0-5.9)	3.2 (2.6-4.0)
HR (95% CI)	1.88 (1.12 to 3.16) *P* = .015		1.24 (0.74 to 2.08) *P* = .42		1.53 (0.90 to 2.59) *P* = .11		—
Non-BM relapse							
Non-BM relapse, No./n	33/950	47/952	32/783	39/787	30/784	41/786	110/2,750
5-year cumulative incidence, % (range)	3.4 (2.4-4.8)	4.7 (3.5-6.3)	3.7 (2.6-5.3)	5.0 (3.7-6.8)	3.7 (2.6-5.3)	5.0 (3.7-6.8)	3.9 (3.3-4.7)
HR (95% CI)	1.43 (0.92 to 2.24) *P* = .11		1.23 (0.77 to 1.96) *P* = .39		1.38 (0.86 to 2.20) *P* = .18		—
Second cancer (as first event)							
SMN, No./n	18/950	4/952	10/783	8/787	3/784	15/786	26/2,750
5-year cumulative incidence, % (range)	1.6 (1.0-2.7)	0.4 (0.2-1.1)	1.3 (0.7-2.4)	0.8 (0.4-1.7)	0.3 (0.1-1.0)	1.8 (1.1-3.0)	0.8 (0.5-1.2)
HR (95% CI)	0.22 (0.07 to 0.65) *P* = .0027		0.80 (0.32 to 2.02) *P* = .63		5.02 (1.45 to 17.35) *P* = .0045		—
Death in CR							
Deaths, No./n	22/942	16/940	9/783	5/787	4/784	10/786	47/2,728
5-year cumulative incidence, % (range)	2.1 (1.3-3.2)	1.4 (0.8-2.4)	0.9 (0.4-1.9)	0.4 (0.1-1.2)	0.4 (0.1-1.2)	0.9 (0.4-1.9)	1.5 (1.1-2.1)
HR (95% CI)	0.73 (0.38 to 1.39) *P* = .34		0.56 (0.19 to 1.67) *P* = .29		2.49 (0.78 to 7.95) *P* = .11		—
Trial treatment-related death^c^							
Deaths, No./n	16/942	11/940	9/783	5/787	4/784	10/786	31/2,728
5-year cumulative incidence, % (range)	1.4 (0.8-2.4)	0.9 (0.4-1.7)	0.9 (0.4-1.9)	0.4 (0.1-1.2)	0.4 (0.1-1.2)	0.9 (0.4-1.9)	0.9 (0.6-1.4)
HR (95% CI)	0.69 (0.32 to 1.49) *P* = .35		0.56 (0.20 to 1.66) *P* = .30		2.49 (0.78 to 7.95) *P* = .11		—

NOTE. All results given in the table are ITT except for the BM relapse pulse random assignment. Rates of induction failure, TRM, and high-risk MRD were compared using Fisher's exact tests; EFS and OS were analyzed using Kaplan-Meier survival analysis with treatment arms compared using Cox regression and the log-rank test. Time to relapse (including site-specific relapse), incidence of second cancer, and trial treatment-related deaths/death in CR were analyzed using competing risks survival analysis by the method of Fine and Grey. For R1 and whole-cohort analyses, times are measured from the date of R1 random assignment/registration and for R2 from the date of R2 random assignment with patients not experiencing an event censored at the date last seen.

Abbreviations: BM, bone marrow; CR, complete remission; EFS, event-free survival; EOI, end of induction; HDM, high-dose methotrexate; HR, hazard ratio; ITT, intention-to-treat; MRD, minimal residual disease; OS, overall survival; R1, randomization 1; R2, randomization 2; SCT, stem cell transplant; SIM, standard interim maintenance; SMN, secondary malignancy; TRM, treatment-related mortality.

^a^ M2 or M3 marrow at EOI, ≥5% MRD at EOI or ≥0.5% at end of consolidation, death from ALL in induction, or trial termination for failure to achieve remission without BM blasts of MRD available. All patients who remained on protocol and were randomly assigned at R2 were induction failures on the basis of MRD (>5%) and all achieved MRD <0.5% by end of consolidation.

^b^ ALL patients only, patients who died of ALL during induction (N = 2) are included as events.

^c^ SCT (in first CR) also treated as a competing risk.

### Randomized Outcomes

#### R1: Short Dex Does Not Reduce Toxicity

Of the 1,902 R1 randomly assigned patients, 1,897 (99.7%) received their randomized arm. Five patients were not given randomized treatment, two in the standard arm (because of error) and three in the short arm (appendicitis, parent choice, and unknown).

**FIG 3.**
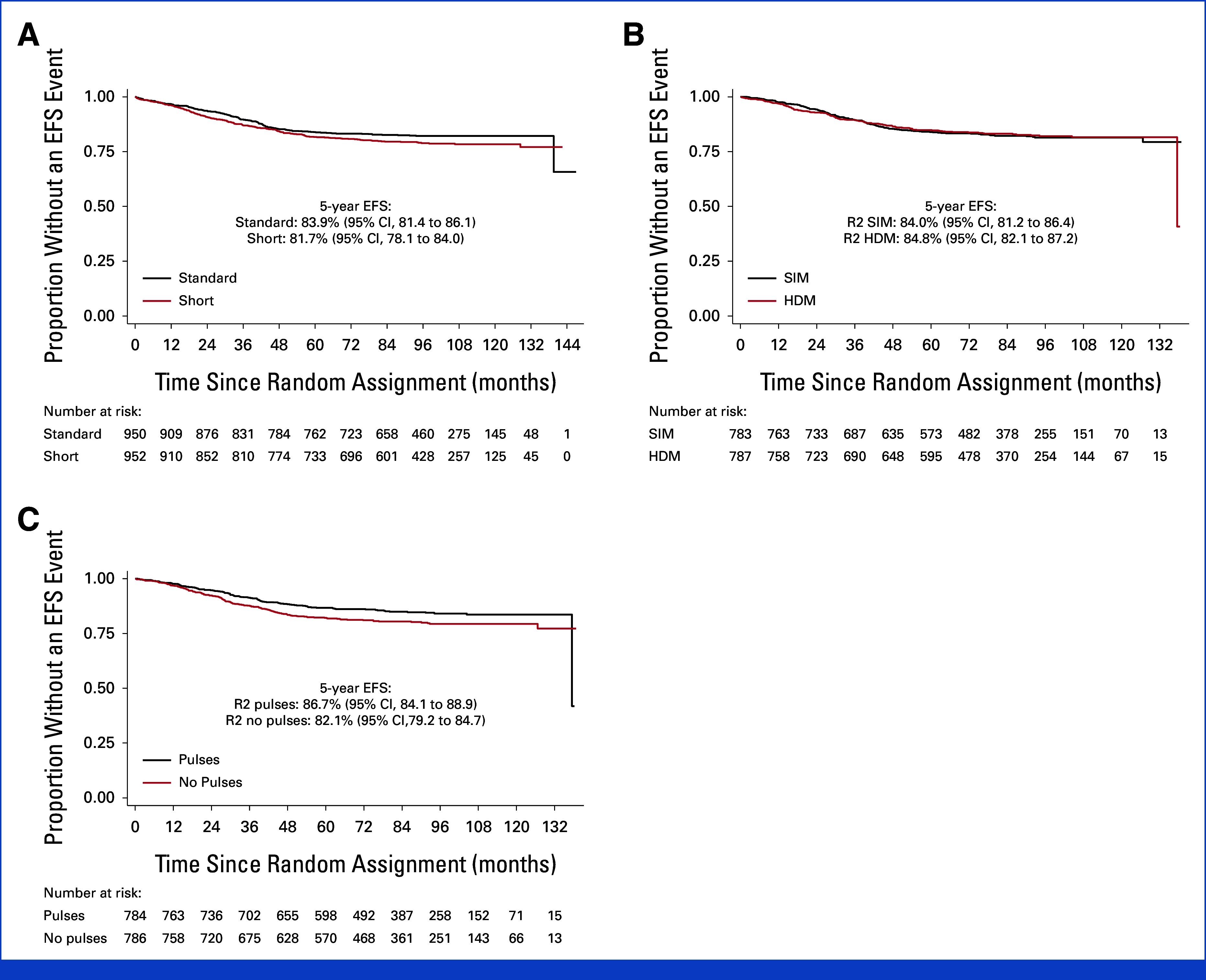
(A) EFS by R1 arms. (B) EFS by R2IM arms. (C) EFS by R2 pulses arms. EFS, event-free survival; HDM, high-dose methotrexate; R1, randomization 1; R2, randomization 2; SIM, standard interim maintenance.

There was no difference in the composite steroid-related toxicity end point between short and standard dex arms (242/950 [25.5%] *v* 227/952 [23.8%], *P* = .41; Table 4), a finding which was consistent within subgroups including age (Data Supplement, Fig S2). Osteonecrosis (ON) rates also did not differ by R1 randomization with 5-year cumulative incidences of 4.3% (95% CI, 3.1 to 5.80) in standard and 3.5% (95% CI, 2.5 to 4.9) in short dex treated patients (HR, 0.82 [95% CI, 0.52 to 1.29]; *P* = .39). Rates of ON were significantly higher in older patients with a 5-year rates of 1.2% (95% CI, 0.7 to 1.9) in under 10s compared with 10.7% (95% CI, 7.8 to 14.7) and 10.4% (95% CI, 7.0 to 15.2) in 10- to 15-year-olds and those age 16 years and older (*P* < .0001, log-rank test for trend), respectively. There were also no differences in early morphologic and MRD response (Data Supplement, Table S1) or EFS between arms (HR, 1.21 [0.98 to 1.49]; *P* = .069; 5-year rates; standard: 83.9% [95% CI, 81.4 to 86.1] *v* short: 81.7% [95% CI, 78.1 to 84.0]; Table 3; Fig 3A).

**TABLE 4.** R1 Steroid Morbidity and Mortality and Selected Toxicities by R2 Arm

Event	Standard (n = 950), No. (%)	Short (n = 952), No. (%)	*P*
R1 random assignment			
Serious steroid AE^a^	107	97	
Grade 3+ dex-related SAE	203	194	
Induction TRM	6	10	
Any event	242 (25.5)	227 (23.8)	.41
Blood and lymphatic system disorders	19 (2.0)	43 (4.5)	
Cardiac disorders	4 (0.4)	0	
GI disorders	36 (3.8)	37 (3.9)	
General disorders and administration site conditions	11 (1.2)	5 (0.5)	
Hepatobiliary disorders	3 (0.3)	0	
Infections and infestations	128 (13.5)	115 (12.1)	
Injury, poisoning, and procedural complications	1 (0.1)	2 (0.2)	
Investigations	12 (1.3)	4 (0.4)	
Metabolism and nutrition disorders	20 (2.1)	20 (2.1)	
Musculoskeletal and connective tissue disorders	9 (1.0)	1 (0.1)	
Nervous system disorders	19 (2.0)	10 (1.1)	
Psychiatric disorders	4 (0.4)	0	
Renal and urinary disorders	5 (0.5)	1 (0.1)	
Respiratory, thoracic, and mediastinal disorders	3 (0.3)	5 (0.5)	
Skin and subcutaneous tissue disorders	1 (0.1)	1 (0.1)	
Vascular disorders	28 (2.9)	13 (1.4)	

Event	SIM (n = 778), No. (%)	HDM (n = 742), No. (%)	*P*
R2 IM random assignment			
Fatal AE	0	0	
Life-threatening or resulted in permeant disability of death^b^	8 (1.0)	10 (1.3)	.56
Any grade 3+ AE	426 (54.8)	392 (52.8)	.47
Renal and urinary disorders	1 (0.1)	17 (2.3)	.0001
Acute kidney injury	1 (0.1)	13 (1.8)	.0007
Pancreatitis	0	6 (0.8)	.013
Creatinine increased	0	5 (0.7)	.028
Blood and lymphatic system disorders	177 (22.8)	136 (18.3)	.036
WBC decreased	119 (15.3)	87 (11.7)	.043

Event	Pulses (n = 769)	No Pulses (n = 739)	*P*
R2 Pulses Random Assignment			
Stopped early due to AEs, No. (%)	9 (1.2)	7 (1.0)	.80
Any SAE, No. (%)	442 (57.5)	411 (55.6)	.47
Fatal SAE, No. (%)^c^	1 (0.1)	1 (0.1)	.98
Life-threatening or resulted in permeant disability of death,^d^ No. (%)	28 (3.6)	31 (4.2)	.58
Number of SAEs, median (IQR)	1 (0-2)	1 (0-2)	.30
Range	0-20	0-15	
Nonfatal AEs grade 3-4	N = 768	N = 739	
Any nonfatal AEs, N (%)	686 (89.3)	619 (83.8)	.0019
Number of AEs, median (IQR)	10 (4-22)	8 (2-18)	<.0001
Febrile neutropenia, No. (%)	460 (59.9)	367 (49.7)	.0001
Median (IQR)	1 (0-2)	0 (0-1)	<.0001
ALT increased, No. (%)	161 (21.0)	104 (14.1)	.0005
Hyperglycemia, No. (%)	11 (1.4)	1 (0.1)	.0063
Platelet count decreased, No. (%)	66 (8.6)	97 (13.1)	.0048
Psychiatric disorders, No. (%)	27 (3.5)	8 (1.1)	.0018
Pain in extremity, No. (%)	8 (1.0)	0	.0077
AST increased, No. (%)	22 (2.9)	5 (0.7)	.0015
Creatinine increased, No. (%)	8 (1.0)	0	.0077

Abbreviations: AE, adverse event; CMV, cytomegalovirus; dex, dexamethasone; HDM, high-dose methotrexate; HLH, hemophagocytic lymphohistiocytosis; IM, interim maintenance; R1, randomization 1; R2, randomization 2; SAE, serious AE; SIM, standard interim maintenance; TRM, treatment-related mortality.

^a^ Serious, grade 3 or AEs related to induction and categorized as steroid-related or steroid-contributory.

^b^ SIM arm: anaphylaxis (n = 2), toxic epidermal necrolysis (n = 1), spinal fracture (n = 1), peripheral motor neuropathy (n = 1), febrile neutropenia (n = 1), weight loss (n = 1), and seizure (n = 1); HDM arm: pancreatitis (n = 1), avascular necrosis (n = 1), seizure (n = 2), allergic reaction (n = 1), sepsis (n = 1), anaphylaxis (n = 1), hemiplegia (n = 1), and encephalopathy (n = 1).

^c^ Pulse arm: CMV pneumonitis (n = 1); no pulse arm: intraoperative splenic injury (n = 1).

^d^ Pulses arm (one patient had two events): avascular necrosis (n = 10), bronchospasm (n = 1), depressed level of consciousness (n = 1), febrile neutropenia (n = 2), fracture (n = 2), headache (n = 1), lethargy (n = 1), lung infection (n = 1), mucosal infection (n = 1), seizure (n = 1), sepsis (n = 3), upper respiratory infection (n = 1), wrist fracture (n = 1), HLH (n = 1), CMV pneumonitis (n = 1), and fungal infection/pneumonia (n = 1). No pulse arm (one patient had five events and one had 2): anaphylaxis (n = 1), osteonecrosis (n = 7), depressed level of consciousness (n = 1), febrile neutropenia (n = 1), fever (n = 2), fracture (n = 2), hypoglycemia (n = 1), hypoxia (n = 1), intracranial hemorrhage (n = 1), intraoperative splenic injury (n = 1), lung infection (n = 1), pneumothorax (n = 1), respiratory failure (n = 1), seizure (n = 5), sepsis (n = 7), stridor (n = 2), tracheal obstruction (n = 1), and tracheitis (n = 1).

#### R2 IM: HDM Does Not Decrease the Risk of CNS Relapse

Fifty (3.2%) R2 randomly assigned patients did not receive their allocated R2IM arm (Fig 1). Of patients given their randomized allocation with returned treatment forms, 103 of 740 (13.9%) did not receive all four doses, with 50 (6.8%) of these due to AEs (most common neurologic N = 15 and renal N = 10). Thirty-nine (5.3%) were switched to SIM after dose 1 or 2 due to AEs and 33 (4.5%) due to delayed clearance without specific AEs reported.

There were 91 CNS relapses (46 standard and 45 HDM) with no difference in CNS relapse rate (CNSR) seen between R2-IM arms; HR, 0.98 (95% CI, 0.65 to 1.49), *P* = .94, 5-year cumulative incidences; SIM 5.3% (95% CI, 3.9 to 7.2); and HDM 5.5% (95% CI, 4.1 to 7.4). No difference was seen in any other time-to-event end points, including EFS; HR, 0.97 (95% CI, 0.77 to 1.24); *P* = .83; 5-year rates: 84.0% (95% CI, 81.2 to 86.4) and 84.8% (95% CI, 82.1 to 87.2; Fig 3B); and OS; HR, 0.89 (0.63 to 1.27); *P* = .53; 93.3% (95% CI, 91.3 to 94.9) and 93.4% (95% CI, 91.4 to 94.9). There was also no difference in CNSR or EFS between arms within prognostic subgroups or by regimen (Data Supplement, Figs S3 and S4).

#### Omission of Maintenance Pulses Is Noninferior for Bone Marrow Relapse

One thousand four hundred thirty of 1,484 patients with ALL were given their randomized allocation, and 54 (3.6%) were not (see Fig 1 for reasons). In the pulses patients who began randomized treatment, dex was given in 6,387 of all 7,343 (87.0%) reported cycles and VCR in 6,885 of 7,387 (93.2%); this equated to 659/761 (86.6%) patients given both dex and VCR in at least 90% of their cycles.

In the per-protocol population, there have been 162 BM relapses (76 pulses and 86 no pulses), giving 5-year cumulative incidence rates of 9.0% (7.1-11.4) and 11.1% (9.0-13.7), an HR of 1.19 (95% CI, 0.87 to 1.62), *P* = .27, and a 5-year difference of +1.7% (–1.5% to 4.1%), that is, falling within the 5% noninferiority margin. In the ITT population, there have been 174 BM relapses (79 in the pulses arm and 95 in the no pulses), giving 5-year cumulative incidences of 9.3% (95% CI, 7.4 to 11.6) and 11.6% (95% CI, 9.5 to 14.1), HR, 1.22 (95% CI, 0.90 to 1.64), *P* = .20, and 5-year difference +1.9% (–1.2% to 4.3%). Although the EFS in the no pulses arm was inferior (per-protocol HR, 1.35 (95% CI, 1.05 to 1.74); *P* = .021; Fig 3C), the difference was smaller and not significant for relapse (per-protocol HR, 1.24 [95% CI, 0.96 to 1.62]; *P* = .10; 5-year difference: +2.8 [–0.6% to 5.5%]). Hence, the effect of omission of pulses on EFS was in part because of an excess of secondary malignancies (SMNs) in that arm (see below). The Data Supplement (Fig S5) shows subgroup analyses for relapse in the pulses randomization with no significant interactions. Note, however, that there was some evidence of a differential effect of pulses by regimen (*P* = .093) with more relapses among patients who received regimen A without pulses (per-protocol difference at 5 years: +5.0 [95% CI, –1.1 to 6.8], 9 of 246 patients with a relapse in the pulses and 22 of 240 in the no pulses group). These were NCI standard-risk and MRD low-risk patients where a similar trend is seen.

#### Interaction Between Arms

The analysis of R2 was confounded by an interaction between R1 and R2IM (*P* = .025 EFS [Data Supplement, Fig S4]; *P* = .018 OS; *P* = .023 relapse). Hence, in the standard R1 arm, HDM had similar or better EFS than SIM, regardless of pulses, but had a worse outcome after short R1 when given without pulses (Data Supplement, Fig S6).

### Toxicity

In the trial overall, 17 patients (0.6%) died from induction treatment–related toxicity (induction TRM) and 47 (5-year cumulative incidence 1.5% [95% CI, 1.1 to 2.1]) died in complete remission (CR; death in CR; Table 3), but no significant differences were seen by R1 or R2 treatment arm. There were two treatment-related deaths in induction and two in remission in 59 patients with Down syndrome. Most treatment-related deaths were due to infection (21/47; 44.7%). Paradoxically, there were more deaths in patients who received three versus four drugs in induction (14/1,463 [1.0%] *v* 3/1,287 [0.2%]; *P* = .025), in large part because of a nonsignificant excess of treatment-related deaths in regimen A patients who received short compared with standard dex (9/503 [1.8%] *v* 4/506 [0.8%]; *P* = .18), with no difference within the NCI high-risk group who received regimen B (four-drug induction; 1/449 [0.2%] *v* 2/444 [0.5%]). However, deaths in CR were more common in older patients with 5-year rates of 1.1% (95% CI, 0.7 to 1.7) for the under 10s and 2.2% (95% CI, 1.2 to 4.0) and 3.3% (95% CI, 1.8 to 6.0) for the 10-15s and 16+ cohorts (*P* = .0008), respectively. To assess deaths related to trial treatment alone, we considered SCT as a competing risk, reducing the rates to 0.9% (95% CI, 0.6 to 1.4) overall and 0.8% (95% CI, 0.5 to 1.4), 0.9% (95% CI, 0.3 to 2.3), and 1.6% (95% CI, 0.7 to 3.9) in the three age groups, respectively (*P* = .12).

Table 4 shows the incidence of selected toxicities by R2 IM and pulses randomizations. Of those given randomized HDM, 439 of 737 (59.6%) had delayed clearance for at least one dose and significantly more patients were reported to have renal toxicity compared with SIM (2.3% *v* 0.1%; *P* = .0001). Pulses were associated with a higher incidence of grade 3-4 AEs, particularly psychiatric disorders, limb pain, hyperglycemia, and febrile neutropenia.

### SMNs

Thirty-two patients were reported to have developed a SMN (Table 3; Data Supplement, Table S1), 26 before relapse, giving a 5-year cumulative incidence of 0.8% (95% CI, 0.5 to 1.2). Within randomized arms (Data Supplement, Table S2), there was an excess of SMNs in standard dex (4 *v* 18), 5-year rates of 0.4% (95% CI, 0.2 to 1.1) and 1.6% (95% CI, 1.0 to 2.7); HR, 0.22 (95% CI, 0.07 to 0.65), *P* = .0027, and no pulses arms (15 *v* 3), 5-year rates: 1.8% (95% CI, 1.1 to 3.0) and 0.3% (95% CI, 0.1 to 1.0); HR, 5.02 (95% CI, 1.45 to 17.35); *P* = .0045, whereas there was no difference between R2IM arms: eight events in HDM, 10 SIM: 5-year rates 1.3% (95% CI, 0.7 to 2.4) and 0.8% (95% CI, 0.4 to 1.7); HR, 0.80 (95% CI, 0.32 to 2.02); *P* = .63.

## DISCUSSION

The 5-year EFS of 83.9% we report here is arguably worse than in our predecessor trial UKALL 2003^3^ (87.2% [95% CI, 85.8 to 88.6]) but with an equivalent 5-year overall survival (92.2% *v* 91.5%). There are several potential reasons for the worse EFS including nonrandomized de-escalation of elements of the chemotherapy backbone, a higher proportion of older patients than in UKALL 2003 (≥16 years 12% *v* UKALL 2003 7%), and the effect of some of the randomized arms on relapse rates. However, a reduction in induction (0.6% *v* 1% UKALL 2003) and remission (1.5% [0.9% censoring for SCT] *v* 2.0% UKALL 2003) TRM is probably a result of the nonrandomized de-escalation of the chemotherapy backbone, in particular the omission of a second delayed intensification. Most striking is the relatively low induction and remission TRM in patients older than 16 years compared with other studies.^7,8^ The similar OS between trials is likely to be due to the lower TRM and better salvage options after relapse, particularly the availability of immune therapy in the current era.

The main aim of UKALL 2011 was to reduce treatment-related morbidity during induction and maintenance and it is disappointing that shortening the duration of dex exposure in induction did not reduce toxicity. Paradoxically, the short dex schedule was associated with a slight excess of treatment-related deaths in younger NCI standard-risk patients who received a three-drug induction. The contrast with older patients, who had a lower induction TRM despite receiving a four-drug induction, is striking, and suggests this is likely to be a chance effect because of the small number of events. Despite the lower TRM with the four-drug regimen, we cannot conclude that an anthracycline-containing induction is safe as there is evidence that it contributes to morbidity without improving survival outcomes.^9^ Although the cumulative exposure to dex in the short arm was lower (140 mg/m^2^ *v* standard 190 mg/m^2^), the higher daily dose in the first 2 weeks of treatment, when patients have poor performance status because of bulk leukemia, may have contributed to the equivalent toxicity with a carry over into the subsequent 2 weeks. On the basis of the observation in the United States Children's Oncology Group (US COG) trial, which used the same 14-day dex schedule, of an excess of ON,^10^ and their previous observation that a week on/week off schedule in delayed intensification reduced the risk of ON,^11^ patients older than 10 years received the discontinuous schedule in our trial. However, we saw no benefit relating to the risk of ON from the discontinuous schedule. Hence, the standard of care within the United Kingdom and Europe (ALLtogether-1 trial) remains the 28-day schedule.

Our other aim was to try and reduce the risk of relapse within the CNS compartment by introducing HDM into our protocol since it had been shown in a relatively recent randomized trial by the US COG^10^ to be of benefit in reducing both CNS and BM relapses in NCI high-risk B-precursor ALL. We found no benefit of HDM in prevention of CNSR or improved EFS including for NCI high-risk patients. However, there were interactions between R1 and R2 with different effects of HDM in R1 arms (Data Supplement, Fig S6). In contrast to the US COG trial,^10^ which showed a benefit of HDM in patients who had received short dex, we found a worse EFS for patients who received that combination, whereas it appeared to be better than SIM in patients who had received standard dex. Omission of pulses led to a worse EFS, although the bone marrow relapse rate difference of +1.7% (–1.5% to 4.1%) was within our prespecified 5% margin for noninferiority, and the effect on relapse rate was smaller and not significantly different. Hence, the EFS difference is likely in large part because of an unexplained excess of SMNs in the no pulses arm. Nevertheless, as there is a potential benefit of pulses on EFS, we cannot conclude that their omission is without increased relapse risk, especially in NCI standard-risk patients who were MRD low-risk and received the lowest-intensity regimen A. Given the excess toxicity of pulses that we report and its potential impact on QoL, whether to give pulses or not will involve weighing the risk and benefits of doing so in an individual patient.

Although the overall rate of SMNs is similar to that reported in other trials,^12^ we saw an excess in the standard dex and no pulses arms for which we have no plausible biologic explanation. Hence, the association is unlikely to be causal.

There has been a recent shift in treatment of BCP ALL toward incorporation of immune therapy such as blinatumomab within existing backbones.^13,14^ Future reductions in toxicity will be obtained by going beyond the cautious de-escalation of chemotherapy backbones, as we tried here, through replacing most if not all of the intensive treatment with these new agents. There is preliminary evidence that this approach is effective in children and young adults with chemotherapy intolerance.^15^ Unfortunately, though, the problem of CNS relapse is less easily solved as there are no new agents in the pipeline that specifically address this issue, apart possibly from CART cell therapy.^16^ More effective CNS-directed therapy will therefore need new targeted or immune therapeutic agents that can better penetrate the CNS. The main limitation of this study is the multiple randomization design which, through unforeseen, possibly chance, interactions, has given results that are difficult to interpret, especially in regard to the subgroup analyses.

In conclusion, although the randomized interventions did not improve outcomes, we report a reduction in TRM compared with previous trials in part due to non-randomized de-escalation of the treatment backbone. Shortening dex exposure during induction does not reduce toxicity and HDM has no benefit for prevention of CNS relapse. Omission of pulses is not associated with a significantly increased risk of relapse, except possibly in patients treated on low-intensity regimens.
